# Time-dependent protective effects of syringic acid following testicular torsion–detorsion: an experimental rat model

**DOI:** 10.1590/acb414226

**Published:** 2026-07-24

**Authors:** Kenan Sabuncu, Mehmet Ali Sezgin, Emre Can Polat, Erkan Merder, Alper Ötünçtemur, Mustafa Baki Çekmen, Saniye Koç Ada, Cem Çomunoğlu, Levent Özcan

**Affiliations:** 1 University of Health Sciences – Prof. Dr. Cemil Tascioglu City Hospital – Department of Urology – Istanbul – Turkey.; 2 Istanbul Medeniyet University – Medical Faculty – Department of Medical Biochemistry – Istanbul – Turkey.; 3 University of Health Sciences – Prof. Dr. Cemil Tascioglu City Hospital – Depatment of Pathology – Istanbul – Turkey.

**Keywords:** Acids, Spermatic Cord Torsion, Reperfusion Injury

## Abstract

**Purpose:**

To investigate the protective effects of syringic acid (SA) against testicular ischemia–reperfusion (I/R) injury during different reperfusion periods in an experimental rat model.

**Methods:**

Forty-eight male Wistar albino rats were randomly assigned to six groups: control, sham, torsion/detorsion (T/D) 4 h, T/D + SA 4 h, T/D 24 h, and T/D + SA 24 h. Testicular torsion was induced by 720° rotation of the left testis for 2 h, followed by detorsion and 4 or 24 h reperfusion. SA (10 mg/kg) was administered intraperitoneally 30 min before detorsion. Oxidative stress markers, histopathological alterations, and immunohistochemical expressions of apoptotic protease activating factor-1 (APAF-1) and inducible nitric oxide synthase were evaluated.

**Results:**

T/D significantly decreased total antioxidant status and glutathione levels while increasing myeloperoxidase activity and APAF-1/inducible nitric oxide synthase (iNOS) expressions compared with controls (*p* < 0.001). SA treatment restored antioxidant capacity and attenuated inflammatory and apoptotic responses (*p* < 0.05). Histopathological analyses demonstrated lower Cosentino scores and higher Johnsen scores in SA-treated groups than in untreated T/D groups (*p* < 0.05). Malondialdehyde levels showed no significant intergroup differences.

**Conclusion:**

SA attenuates testicular I/R injury by reducing oxidative stress, inflammation, and apoptosis while preserving spermatogenic function and histological integrity.

## Introduction

Testicular torsion is a major urological emergency with a peak incidence observed in newborns, children, and adolescents^1^. Even after successful detorsion, temporary ischemia may cause permanent testicular damage, including apoptosis, atrophy, impaired spermatogenesis, and potential infertility. Therefore, rapid diagnosis and intervention are essential^2,3^. Testicular ischemia–reperfusion (I/R) injury is characterized by excessive reactive oxygen species (ROS) production, lipid peroxidation, inflammatory responses, and germ-cell apoptosis, all of which contribute to impaired spermatogenesis and testicular dysfunction. Previous experimental studies have demonstrated that oxidative stress significantly disrupts sperm quality, antioxidant enzyme activity, and reproductive hormone balance in testicular tissue. Furthermore, various antioxidants have been shown to attenuate oxidative damage by reducing malondialdehyde (MDA) levels and restoring endogenous antioxidant defense mechanisms. In this regard, β-cryptoxanthin has been reported to ameliorate testicular I/R injury by improving histopathological architecture, decreasing oxidative stress markers, and preserving spermatogenic activity in a rat torsion–detorsion model^4,5^. Consequently, various medical therapies are being investigated to mitigate I/R injury alongside surgical management.

Polyphenolic compounds exert protective effects against oxidative tissue injury through free radical scavenging, suppression of lipid peroxidation, and regulation of cellular antioxidant pathways. Accumulating experimental evidence has shown that polyphenol-rich natural extracts improve reproductive outcomes and modulate oxidative stress markers, including Nrf2 and inducible nitric oxide synthase (iNOS) expression, in testicular injury models^6^. Syringic acid (SA), a naturally occurring phenolic acid present in various fruits and medicinal plants, has recently attracted increasing attention due to its potent antioxidant, anti-inflammatory, and anti-apoptotic effects^7,8^.

Experimental studies have demonstrated that SA attenuates I/R-induced tissue injury by suppressing lipid peroxidation, restoring endogenous antioxidant enzyme activity, and modulating oxidative stress-related signaling pathways^8^. Furthermore, recent evidence suggests that the protective effects of SA may involve suppression of the HMGB1/NF-κB axis, attenuation of endoplasmic reticulum stress, and inhibition of apoptosis-related pathways such as caspase-3 activation^7^. When compared to traditional antioxidant agents studied in testicular torsion models, SA may provide broader cytoprotective effects through the simultaneous modulation of pathways related to oxidative stress, inflammatory signaling, endoplasmic reticulum stress, and apoptosis.

Although previous experimental studies have demonstrated the protective effects of SA against testicular torsion-detorsion injury by reducing oxidative stress, inflammation, and apoptosis, many of the mechanistic aspects of its tissue-protective activity remain poorly understood. To date, studies have primarily focused on conventional oxidative stress and histopathological parameters. However, data regarding apoptosis-related pathways, nitric oxide-associated oxidative damage, and the effects of different reperfusion durations remain limited^7,9^. Furthermore, comprehensive evaluations integrating biochemical, histopathological, and immunohistochemical findings during both early and late reperfusion periods remain scarce in the current literature.

Therefore, this study aimed to investigate the protective effects of SA against testicular torsion-detorsion injury at different reperfusion times by evaluating oxidative stress parameters, histopathological changes, and the immunohistochemical expression of apoptotic protease activating factor-1 (APAF-1) and iNOS in experimental rat models.

## Methods

The study was conducted following approval of the animal research protocol (No. 2023/02) by the Local Ethics Committee for Animal Experiments at the Istanbul Mehmet Akif Ersoy Experimental Research, Development, and Training Center. Subsequently, a total of 48 adult male Wistar albino rats, aged 12 weeks old and weighing 350–400 g, were obtained from the same center. No formal power analysis was performed, but the number of animals included in each group (n = 8) was determined according to previous experimental studies evaluating I/R injury and antioxidant treatment protocols in rat testicular torsion models^9^.

Rats were acclimated to the new environment for seven days before starting the experimental procedures. Rats were housed in groups of three or four per cage. Throughout the experiment, the animals were maintained under pathogen-free conditions in ventilated rooms at 24°C, with a 12-hour light/12-hour dark cycle, and were provided food and water *ad libitum*. The surgical area of all animals was prepared using a 10% povidone-iodine solution prior to all procedures. All procedures were performed in accordance with the Animal Research: Reporting of In Vivo Experiments (ARRIVE) guidelines.

### Surgical procedure

For anesthesia, all rats were administered intraperitoneal ketamine hydrochloride (50 mg/kg) and xylazine hydrochloride (10 mg/kg). After adequate anesthesia was achieved, the surgical field was sterilized and draped under aseptic conditions. The left testis was exteriorized through a median raphe scrotal incision and rotated 720° clockwise to induce testicular torsion. The testis was fixed to the scrotum with a 4/0 prolene suture, and the incision was closed. After 2 h of torsion, detorsion was performed by returning the testis to its normal anatomical position. At the end of the experimental period, all rats were sacrificed under general anesthesia.

### Groups and drug administration

Rats were randomly assigned to six experimental groups using a simple randomization procedure, with each group consisting of eight rats:

- Group 0 (sham group): A scrotal incision was made to expose the testicle. The testicle was then fixed to the scrotum with 4-0 vicryl sutures without performing a torsion procedure. Orchiectomy was performed 30 minutes later;
- Group 1 (control group): Orchiectomy was performed without induction of testicular torsion or SA administration;
- Group 2 (T/D-early group): Testicular torsion was induced for 2 h, followed by detorsion and 4 h of reperfusion before orchiectomy^10^;
- Group 3 (T/D+SA-early group): The same surgical procedure as in group 2 was performed. In addition, SA (10 mg/kg; Medchem, United States of America), dissolved in physiological saline, was administered intraperitoneally 30 min before detorsion.
- Group 4 (T/D-delayed group): Testicular torsion was induced for 2 h, followed by detorsion and 24 h of reperfusion before orchiectomy;
- Group 5 (T/D+SA-delayed group): The same surgical procedure as in group 4 was performed. In addition, SA (10 mg/kg; Medchem, United States of America), dissolved in physiological saline, was administered intraperitoneally 30 min before detorsion.

In groups 3 and 5, SA was administered 30 min before detorsion to ensure adequate systemic bioavailability prior to the onset of reperfusion. This timing was preferred because the major component of testicular in I/R injury occurs during the reperfusion phase through excessive reactive oxygen species generation and inflammatory activation^7^.

Histopathological and immunohistochemical assessments were performed by a pathologist blinded to group allocation.

### Dosage

For the biological effects of SA (Medchem, United States of America, Catalog Number: HY-N0339-200), the dosage was determined as 10-mg/kg body weight based on previous studies in the literature^8,11^.

### Biochemical analysis

A segment of the testicular tissue was immersed in Bouin’s solution for the purpose of histopathological examination, while the residual portion was stored at -80°C for the purpose of oxidative stress analysis. Tissue levels of malondialdehyde (MDA), reduced glutathione (GSH), myeloperoxidase (MPO), and total antioxidant status (TAS) were measured.

### Sample preparation

A quantity of tissue, ranging from 250 mg to 300 mg, was homogenised in 10 volumes of cold 0.15% potassium chloride (KCl) solution using an Ultra-Turrax homogenizer. The homogenates were centrifuged at 1,600 × g for 10 min at 4°C, and the supernatants were collected and stored at -80°C until biochemical analysis.

### Lipid peroxidation and antioxidant analysis

MDA levels were measured spectrophotometrically at 532 nm after incubation of tissue homogenates with thiobarbituric acid (TBA) and trichloroacetic acid (TCA), as previously described in oxidative stress studies^12,13^.

Reduced GSH levels were determined by reacting the supernatant with sodium phosphate buffer (Na_2_HPO_4_) and 5,5’-dithiobis-(2-nitrobenzoic acid) (DTNB), followed by spectrophotometric analysis at 412 nm^13,14^.

### Myeloperoxidase measurement

Homogenates were centrifuged at 18,000 × g for 15 min at 4°C. Subsequently, 10 µL of supernatant was mixed with 290 µL of reagent solution. Absorbance was measured spectrophotometrically at 460 nm at 0 and 60 s. MPO activity was determined based on the oxidation of o-dianisidine dihydrochloride in the presence of hydrogen peroxide (H_2_O_2_).

### Total antioxidant status measurement

TAS levels were measured using a commercial assay kit (Elabscience, E-BC-K801M) according to the manufacturer’s instructions. Absorbance was measured spectrophotometrically at 660 nm, and the results were expressed as Trolox equivalents.

### Histopathological and immunohistochemical analysis

Testicular tissues were subjected to staining with hematoxylin-eosin (HE) and examined under a light microscope. Spermatogenesis was evaluated using the Johnsen testicular biopsy scoring (JTBS) system by analysing 50 seminiferous tubules per sample^15^. The Cosentino scoring system was used to determine the degree of testicular ischaemic failure and injury^16^.

The tissue samples were processed on an automated tissue processing device (Diapath) and embedded in paraffin at temperatures ranging from 56 to 58°C. Four-µm-thick sections from paraffin blocks were prepared using a microtome and mounted on slides. To HE staining, the sections were subjected to a series of xylene and alcohol washes, followed by staining with HE. Finally, the sections were mounted using Entellan. Two slides were prepared from each sample and evaluated by a pathologist using an Olympus BX53 microscope.

Immunohistochemical analyses were conducted to examine the expression levels of APAF-1 and iNOS. Antigen retrieval was performed in citrate buffer (pH 6.0) using heat-induced epitope retrieval, followed by blocking of endogenous peroxidase activity with hydrogen peroxide solution. Sections were then incubated with APAF-1 (1:200; BIOSS, bs-0058R) and iNOS (1:200; BIOSS, bs-2072R) primary antibodies. The staining process was conducted utilising a secondary antibody (ROCHE/VENTANA) and DAB chromogen, while contrast staining was performed with Mayer hematoxylin. It should be noted that the primary antibody was not included in the negative controls. The intensity of immunostaining was then assigned a score ranging from 0 (absent) to 3 (strong).

### Statistical method

Statistical analyses were performed using Statistical Package for the Social Sciences version 27.0 (IBM Corp., Armonk, NY, United States of America). Data distribution was evaluated using the Shapiro–Wilk and Kolmogorov–Smirnov’s tests. Continuous variables were expressed as mean ± standard deviation or median (minimum–maximum), as appropriate. Intergroup comparisons were performed using the Kruskal–Wallis’ test followed by Mann–Whitney’s U tests for pairwise comparisons. Categorical variables were analyzed using the χ^2^ test. A *p* < 0.05 was considered statistically significant.

## Results

### Oxidants and antioxidants

GSH levels were significantly decreased in the T/D groups compared with the sham and control groups. Administration of SA significantly increased GSH levels in the treatment groups. GSH levels in groups 3 and 5 were significantly higher than those in groups 2 and 4, respectively (*p* < 0.05). Although GSH levels were higher in the 24-h reperfusion SA-treated group than in the early reperfusion SA-treated group, the difference was not statistically significant.

MPO levels were significantly higher in the T/D groups than in the sham and control groups. Group 4 showed significantly higher MPO levels than group 5 (*p* < 0.05).

TAS levels in group 4 were significantly lower than those in groups 0, 1, and 5 (*p* < 0.05). No statistically significant differences were observed in MDA levels among the groups (Table 1).

**Table 1:** Biochemical parameters in testicular tissues of experimental groups<tfn href="tfn01">*</tfn>.

	Group 0	Group 1	Group 2	Group 3	Group 4	Group 5	*p* -value
GSH (µmol/g tissue)	7.2 ± 1.5	8.0 ± 0.8	6.3 ± 0.4	8.7 ± 1.9	5.9 ± 1.0	9.7 ± 1.6	< 0.05
MPO (U/g tissue)	0.8 ± 0.2	0.5 ± 0.2	1.1 ± 0.4	1.5 ± 0.7	2.8 ± 1.3	1.7 ± 0.7	< 0.05
TAS (mmol Trolox equivalent/g tissue)	10.8 ± 1.9	9.2 ± 4.2	7.0 ± 2.1	7.0 ± 2.8	4.2 ± 1.2	8.1 ± 3.5	< 0.05
MDA (nmol/g tissue)	43.6 ± 3.7	43.5 ± 8	47.1 ± 6.4	44.2 ± 2.5	45.3 ± 4.7	41.2 ± 2.8	> 0.05

* Data are presented as mean ± standard deviation. Statistical analyses were performed using the Kruskal–Wallis test followed by the Mann–Whitney U test; GSH: reduced glutathione; MPO: myeloperoxidase; TAS: total antioxidant status; MDA: malondialdehyde.

Source: Elaborated by the authors.

### Histopathological evaluation

Cosentino scores were significantly higher in the torsion groups (groups 2 and 4) compared with the sham, control, and treatment groups. Although lower Cosentino scores were observed in the SA-treated groups (groups 3 and 5) compared with the untreated torsion groups, the differences were not statistically significant (Table 2).

**Table 2:** Histopathological evaluation scores in experimental groups<tfn href="tfn01">*</tfn>.

	Group	Median	Min	Max	p-value
**Cosentino**	Group 0	1	1	2	< 0.001
	Group 1	1	1	1	
	Group 2	4	3	4	
	Group 3	2	2	3	
	Group 4	4	4	4	
	Group 5	2	2	3	
	**Group**	**Median**	**Min**	**Max**	**p-value**
**JTBS**	Group 0	9.4	8.4	9.6	< 0.001
	Group 1	9.8	9.6	9.9	
	Group 2	8.5	8	9.3	
	Group 3	9.2	8	9.7	
	Group 4	8	3.4	9.1	
	Group 5	9.2	9.1	9.7	

* Data are presented as median (minimum–maximum). Statistical analyses were performed using the Kruskal–Wallis test followed by the Mann–Whitney U test for pairwise comparisons; JTBS: Johnsen testicular biopsy scoring.

Source: Elaborated by the authors.

JTBS values were significantly higher in the sham and control groups (groups 0 and 1) than in the torsion groups. In addition, groups 3 and 5, which received SA treatment, showed significantly higher JTBS scores compared with groups 2 and 4, respectively (Table 2).

**Figure 1:**
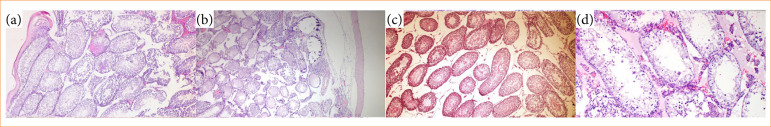
Intergroup Johnsen and Cosentino scores and stainings: (a) sham group Johnsen score 8, 9 and 10 tubule structures (HEx40); (b) group 4 Johnsen score 1, 2, 3 and 4 tubule structures (HEx40); (c) control group normal area (HEx40); (d) group 5 Cosentino score 2 area (HEx160).

Microscopic images of testicular tissues are presented in Fig. 1.

HE: hematoxylin and eosin. Source: Elaborated by the authors.

### Immunohistochemical analysis

The highest APAF-1 and iNOS immunoreactivity scores were observed in the torsion groups (groups 2 and 4) (*p* < 0.05). Groups 2 and 4 demonstrated significantly higher APAF-1 and iNOS expression levels compared with the sham, control, and SA-treated groups. Intense iNOS immunostaining was detected in groups 2 and 4. In contrast, APAF-1 and iNOS expression levels were significantly lower in the SA-treated groups (groups 3 and 5) than in the untreated torsion groups (groups 2 and 4). No statistically significant differences were observed between the SA-treated groups and the sham/control groups regarding APAF-1 and iNOS immunoreactivity (Tables 3 and 4).

**Table 3:** Distribution of inducible nitric oxide synthase (iNOS) and apoptotic protease activating factor-1 (APAF-1) immunoreactivity scores in experimental groups<tfn href="tfn01">*</tfn>.

i-NOS	+			++			+++			++++		*p* -value
	n	%		n	%		n	%		n	%	
Group 0	5	62.5		3	37.5		0	0		0	0	< 0.001
Group 1	6	75		2	25		0	0		0	0	
Group 2	0	0		0	0		1	12.5		7	87.5	
Group 3	0	0		6	75		2	25		0	0	
Group 4	0	0		0	0		0	0		8	100	
Group 5	0	0		7	87.5%		1	12.5		0	0	
**APAF-1**	**+**			**++**			**+++**			**++++**		***p* -value**
	**n**	**%**		**n**	**%**		**n**	**%**		**n**	**%**	
Group 0	6	75		2	25		0	0		0	0	< 0.001
Group 1	7	87.5		1	12.5		0	0		0	0	
Group 2	0	0		0	0		2	25		6	75	
Group 3	0	0		5	62.5		3	37.5		0	0	
Group 4	0	0		0	0		1	12.5		7	87.5	
Group 5	0	0		6	75 %		2	25		0	0	

* Data are presented as number (percentage). Statistical analyses were performed using the χ^2^ test.

Source: Elaborated by the authors.

**Table 4:** Inducible nitric oxide synthase (iNOS) and apoptotic protease activating factor-1 (APAF-1) immunoreactivity scores in experimental groups*.

	Group	Median	Min	Max	p-value
**iNOS**	Group 0	0	0	1	< 0.001
	Group 1	0	0	1	
	Group 2	3	2	3	
	Group 3	1	1	2	
	Group 4	3	3	3	
	Group 5	1	1	2	
	**Group**	**Median**	**Min**	**Max**	**p-value**
**APAF-1**	Group 0	0	0	1	< 0.001
	Group 1	0	0	1	
	Group 2	3	2	3	
	Group 3	1	1	2	
	Group 4	3	2	3	
	Group 5	1	1	2	

* Data are presented as median (minimum–maximum). Statistical analyses were performed using the Kruskal–Wallis’ test followed by the Mann–Whitney’s U test for pairwise comparisons.

Source: Elaborated by the authors.

**Figure 2:**
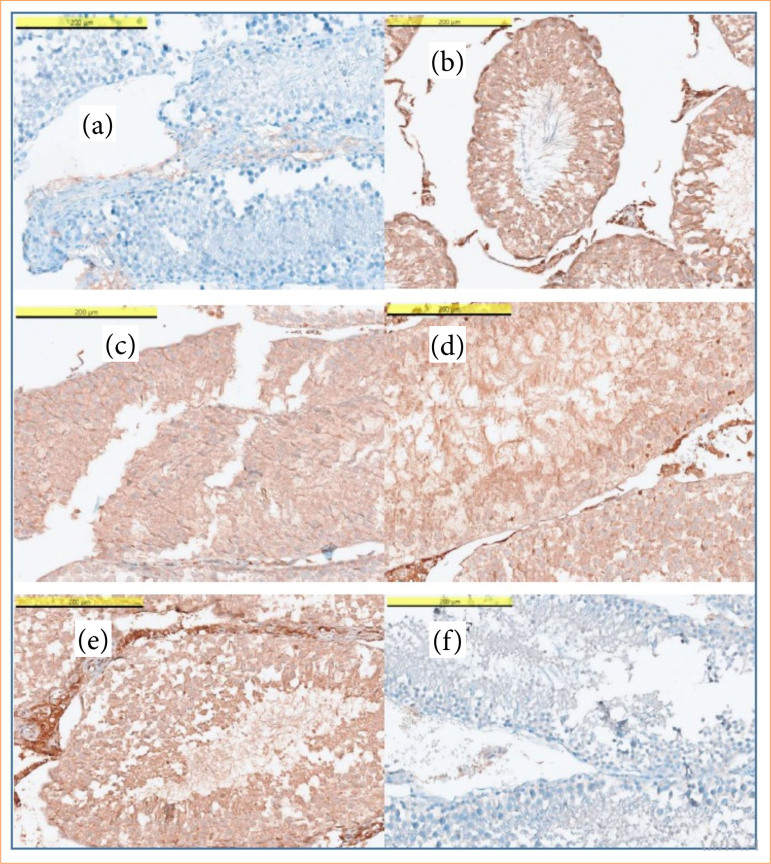
Apoptotic protease activating factor-1 (APAF-1) and inducible nitric oxide synthase (i-NOS) staining levels among the groups: (a) group 1, APAF negative (x400); (b) group 3, APAF-1 weak staining (x400); (c) group 2, APAF-1 moderate staining (x400); (d) group 4, APAF-1 strong staining (x400); (e) group 3, i-NOS moderate staining (x400); (f) group 4, i-NOS strong staining (x400).

Source: Elaborated by the authors.

Microscopic images of testicular tissues of the groups are presented in Fig. 2.

## Discussion

The present study demonstrated that SA attenuated testicular I/R injury by improving oxidative stress parameters, reducing inflammatory and apoptotic responses, and preserving histopathological architecture in a rat torsion–detorsion model.

The testes are particularly vulnerable to oxidative stress because of their high content of polyunsaturated fatty acids and continuous spermatogenic activity. During testicular I/R injury, excessive reactive oxygen species (ROS) production induces lipid peroxidation, inflammatory activation, and cellular damage, ultimately impairing testicular function. Previous studies have demonstrated that ROS overproduction during the ischemia and reperfusion phases promotes leukocyte migration, inflammatory cytokine release, lipid peroxidation, and apoptotic cell injury in testicular tissue^17-20^. A recent experimental study investigating Mito-TEMPO in testicular torsion/detorsion injury similarly reported that oxidative stress-mediated damage was associated with increased MDA levels and impaired antioxidant enzyme activity, further supporting the central role of oxidative stress in testicular I/R injury^20^.

TAS reflects the cumulative antioxidant capacity of biological tissues. In the present study, decreased TAS levels in the torsion/detorsion (T/D) groups further supported the contribution of oxidative stress to testicular I/R injury. The partial restoration of TAS levels following SA administration may be associated with the antioxidant and free radical scavenging properties of SA. Similarly, reduced GSH, one of the major endogenous antioxidant defense molecules, was significantly decreased in the T/D groups, indicating impaired antioxidant capacity. SA treatment increased GSH levels, suggesting that SA may contribute to the preservation of endogenous antioxidant defense mechanisms.

Inflammation has been identified as another important mechanism contributing to testicular damage following T/D injury^19^. Increased MPO levels observed in the T/D groups in the present study indicate enhanced neutrophil infiltration and inflammatory activation secondary to I/R injury. Treatment with SA significantly reduced MPO levels compared with the untreated torsion groups, suggesting that SA may attenuate inflammatory responses associated with testicular I/R injury. Similar protective effects have also been reported for other natural antioxidant agents. Behnejad et al. demonstrated that allicin and hesperidin reduced oxidative stress and improved testicular tissue integrity by enhancing antioxidant defense mechanisms in experimental testicular injury models^21^.

MDA is a widely used marker of lipid peroxidation and oxidative tissue injury. Previous experimental studies investigating testicular I/R injury have generally reported increased MDA levels following T/D^19,22,23^. While some studies demonstrated statistically significant elevations in MDA levels^19^, other studies using different ischemia and reperfusion durations reported no significant intergroup differences^24,25^. In the present study, MDA levels were numerically higher in the torsion groups and lower in the SA-treated groups, but these differences did not reach statistical significance. Since MDA levels may vary depending on the severity and timing of oxidative injury, the selected reperfusion periods and the limited sample size may have influenced the statistical outcomes. In addition, methodological differences such as SA dose, route of administration, and torsion–reperfusion duration may partially explain the discrepancy between our findings and previous studies reporting significant reductions in MDA levels following SA treatment^7,9^.

Nitric oxide (NO) is a short-lived free radical synthesized from L-arginine via nitric oxide synthase (NOS) enzymes^26^. Increased NOS activity under I/R conditions triggers peroxynitrite formation and contributes to cellular damage^27^. Oxidative stress and DNA damage developing in I/R injury increase mitochondrial membrane permeability, leading to activation of the APAF-1/caspase-9 pathway and initiation of intrinsic apoptosis^28^. In a recent experimental study, fisetin-based antioxidant therapy was also shown to attenuate oxidative stress and apoptotic pathways in testicular I/R injury, supporting the role of antioxidant-mediated protection against testicular damage^29^.

In our study, APAF-1 and iNOS levels were found to be significantly higher in the T/D groups compared to the control groups (*p* < 0.05), while these levels were significantly decreased in the SA-treated groups compared to the T/D groups. These findings suggest that SA may exert anti-inflammatory and anti-apoptotic effects in testicular I/R injury by suppressing the iNOS-mediated inflammatory response and the APAF-1/caspase pathway. Similar reductions in apoptosis-related signaling pathways have also been reported in recent antioxidant-based experimental studies of testicular I/R injury^29^.

Previous studies investigating experimental testicular torsion models have demonstrated that prolonged ischemia and reperfusion periods increase testicular damage, resulting in higher Cosentino scores and lower JTBS^2,16,30-32^. In the present study, Cosentino scores were significantly lower in the sham and control groups (*p* < 0.05). Although Cosentino scores numerically increased with longer reperfusion periods, the difference between reperfusion groups did not reach statistical significance. This finding may be associated with the relatively shorter reperfusion durations used in our experimental model compared with previous studies. In contrast, JTBS appeared to reflect earlier histopathological alterations associated with germ cell loss and apoptosis during I/R injury. SA-treated groups demonstrated improved Cosentino and JTBS scores compared with the untreated torsion groups, suggesting a protective effect of SA against testicular tissue injury. Similar histopathological improvements following antioxidant treatment have also been reported in recent experimental studies of testicular I/R injury^33-36^.

Previous experimental studies^7,9^ demonstrated the protective effects of SA against I/R injury following testicular torsion, but these studies evaluated only a single reperfusion period and therefore provided limited information regarding the temporal progression of tissue injury after detorsion. In the present study, two different reperfusion intervals (4 and 24 h) were included to represent different phases of I/R injury. This approach enabled evaluation of both the early and relatively later effects of SA on oxidative stress, inflammation, apoptosis, and histopathological damage. Although additional experimental and long-term studies are needed, our findings suggested that SA may maintain its protective effects beyond the acute phase of injury.

Several limitations should be considered when interpreting these findings. The study was conducted in an experimental animal model with a relatively limited sample size. In addition, long-term functional outcomes such as fertility potential, hormonal recovery, and spermatogenic capacity were not evaluated. The effects of SA beyond the selected reperfusion periods also remain unclear. Another limitation of the present study was the absence of a SA-only group. Nevertheless, the primary aim of the study was to evaluate the protective effects of SA against I/R injury rather than its isolated physiological effects in normal testicular tissue. Furthermore, unnecessary expansion of experimental groups was avoided in accordance with the principles of reduction in animal research.

## Conclusion

SA demonstrated protective effects against testicular I/R injury in this experimental rat model by improving oxidative stress parameters, reducing inflammatory and apoptotic responses, and preserving histopathological architecture. These findings suggested that SA may contribute to attenuation of testicular damage following T/D injury. Further experimental studies are needed to clarify its long-term effects and underlying mechanisms.

## Data Availability

The datasets used and/or analysed during the current study are available from the corresponding author on reasonable request.
